# Water-Soluble Epitaxial NaCl Thin Film for Fabrication of Flexible Devices

**DOI:** 10.1038/s41598-017-09603-5

**Published:** 2017-08-18

**Authors:** Dong Kyu Lee, Sungjoo Kim, Sein Oh, Jae-Young Choi, Jong-Lam Lee, Hak Ki Yu

**Affiliations:** 1 Dept. of Materials Science and Engineering & Dept. of Energy Systems Research, Ajou University, Suwon, 16499 Korea; 2 Dept. of Materials Science and Engineering, Pohang University of Science and Technology, Pohang, 37673 Korea; 3 School of Advanced Materials Science & Engineering, Sungkyunkwan University, Suwon, 16419 Korea

## Abstract

We studied growth mechanisms of water-soluble NaCl thin films on single crystal substrates. Epitaxial growth of NaCl(100) on Si(100) and domain-matched growth of NaCl(111) on c-sapphire were obtained at thicknesses below 100 nm even at room temperature from low lattice mismatches in both cases. NaCl thin film, which demonstrates high solubility selectivity for water, was successfully applied as a water-soluble sacrificial layer for fabrication of several functional materials, such as WO_3_ nano-helix and Sn doped In_2_O_3_ nano-branches.

## Introduction

Recently, research directed at achieving flexibility and functionality in displays, sensors, and photocatalytic devices has attracted considerable interest because of the development in the fields of organic materials and nanotechnology^1–3^. The flexible substrate supporting the functional device, is the most critical component for development of ultimate flexible devices^4^. Until now, several materials have been studied as flexible substrates, including plastic materials such as PET (polyethylene terephthalate) and PEN (polyethylene naphthalate), as well as inorganic materials, such as metal foil and thin glass^5–10^. Considering ultimate curvature, device process, convenience, and productivity, plastic is the most suitable material for flexible substrates^11^. However, plastic substrates cannot withstand high processing temperatures and hence, their applications are limited^12^.

**Figure 1:**
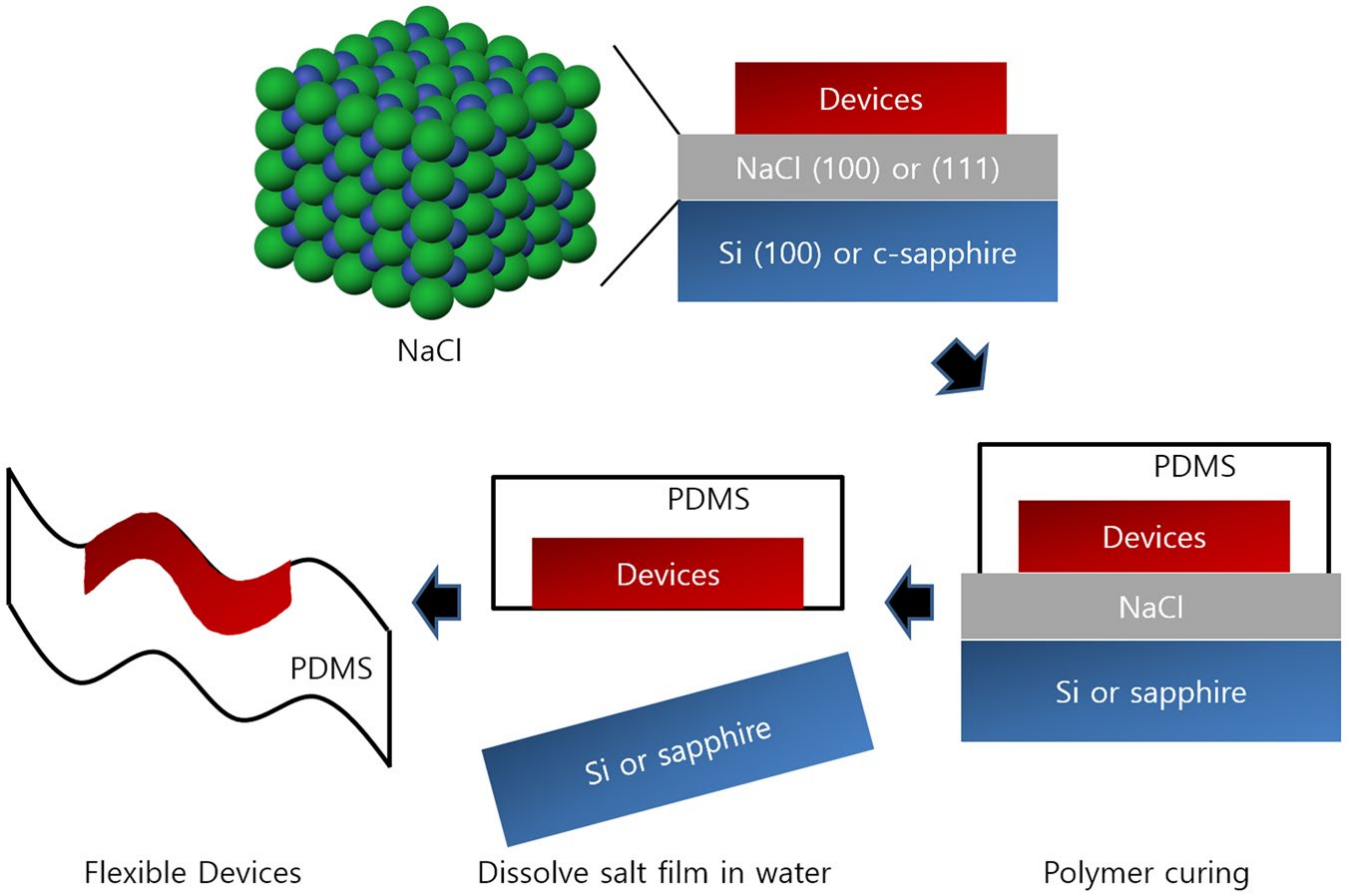
Schematic showing the experimental procedure for the fabrication of flexible devices based on water-soluble NaCl sacrificial layer.

Herein, we suggest a new method for fabrication of flexible devices using water-soluble inorganic thin films. After growing water-soluble thin film on general substrates, such as silicon and glass, high-temperature processing may be conducted without damaging the substrate because of high melting point of inorganic material^13^. A polymer adhesive, such as PDMS (polydimethylsiloxane) and a UV (ultra-violet) curable bonding agent may be used to prepare the flexible substrate. Finally, the flexible device may be obtained after dissolving inorganic thin film in water as shown in schematics in Fig. 1 ^14^. We used NaCl (sodium chloride) as the water-soluble buffer layer for fabrication of the flexible device and analyzed its growth mechanism. The solubility selectivity of NaCl depends on the solvent. It dissolves more quickly in water (360 g NaCl/1 kg solvent), than in other solvents, such as methanol (14 g/1 kg), ethanol (0.65 g/1 kg), and acetone (0.00042 g/1 kg)^15^. NaCl can endures high processing temperatures because its melting point is approximately 801 °C^16^. Lattice mismatch between NaCl and conventional Si substrates is only 3.8%, and hence, it may be grown epitaxially on Si substrates^17^. Using the proposed method, many functional materials requiring high-temperature processing may be used for fabrication of flexible devices by controlling orientation of the NaCl buffer layer. For example, perovskite (ABO_3_) materials exhibiting ferromagnetic or ferroelectric properties may be grown on NaCl(100) films, and functional materials with hexagonal close-packed crystal structure, such as GaN, ZnO, and MaB_2_ may be prepared on NaCl(111) films^18–20^.

In this study, we examined growth mechanisms of NaCl films on Si(100) and c-plane sapphire substrates. NaCl films were grown by thermal evaporation (the growth rate was −1 nm/s, and substrate temperature was maintained at room temperature for commercial applications) rather than the effusion cell method (the conventionally used method). We used XRD (X-ray diffraction) to identify the growth mode of NaCl films with respect to film thickness. From experimental results, we proposed mechanisms for epitaxial growth of NaCl films on Si(100) and c-sapphire substrates. In addition, we demonstrated the possibility of transferring large-area functional nanomaterials (greater than 4 inches) by dissolving the NaCl buffer layer in pure water using WO_3_ nano-helix. We tested the effect of NaCl crystallinity on electrical properties of functional nano-materials (Sn doped In_2_O_3_ nano-branches: ITO NB) during transfer process.

## Results and Discussion

**Figure 2:**
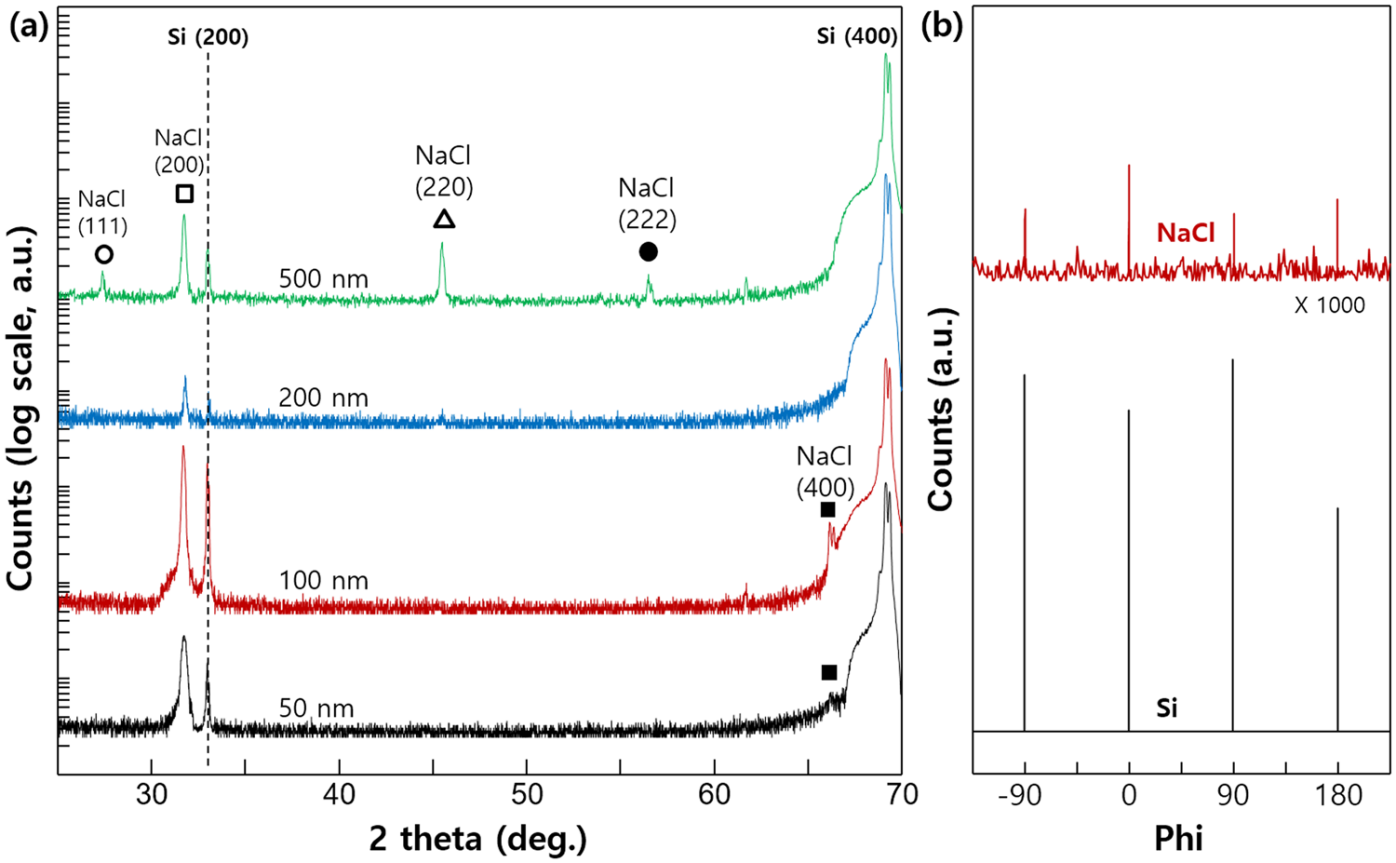
(**a**) XRD patterns of NaCl films grown on Si(100) substrates at different film thicknesses (**b**) Azimuthal diffraction scans across the off-normal NaCl $\{2\bar{2}0\}$ and Si $\{1\bar{1}0\}$ for 100 nm-thick NaCl film on Si(100) substrate.

XRD patterns of NaCl films grown on Si(100) substrates at different film thicknesses are shown in Fig. 2a. For film thicknesses up to 100 nm, only the NaCl(200) peak was observed in XRD data. When film thickness was greater than 200 nm, intensity of the NaCl(200) peak decreased, and the NaCl(111) peak was observed. Considering the intensity ratio of (111) and (200) diffraction peaks per the JCPDS (Joint Committee on Powder Diffraction Standards) file (05–0628), i.e., 13 for (111) and 100 for (200), the preferred orientation is NaCl(111) for thick films (thickness ≥500 nm). Phi-scan (azimuthal scan) analyses across the off-normal NaCl $\{2\bar{2}0\}$ and Si $\{1\bar{1}0\}$ were also conducted to validate epitaxial relationship between NaCl film (100 nm thick) and Si(100) substrate; results are presented in Fig. 2b. Coincidence in angular positions of Si $\{1\bar{1}0\}$ and NaCl $\{2\bar{2}0\}$ reflections indicate these two zone axes are aligned. With crystal symmetry considered, we determined in-plane epitaxial relation to be $(200){[2\bar{2}0]}_{NaCl}\parallel (100){[1\bar{1}0]}_{Si}$.

**Figure 3:**
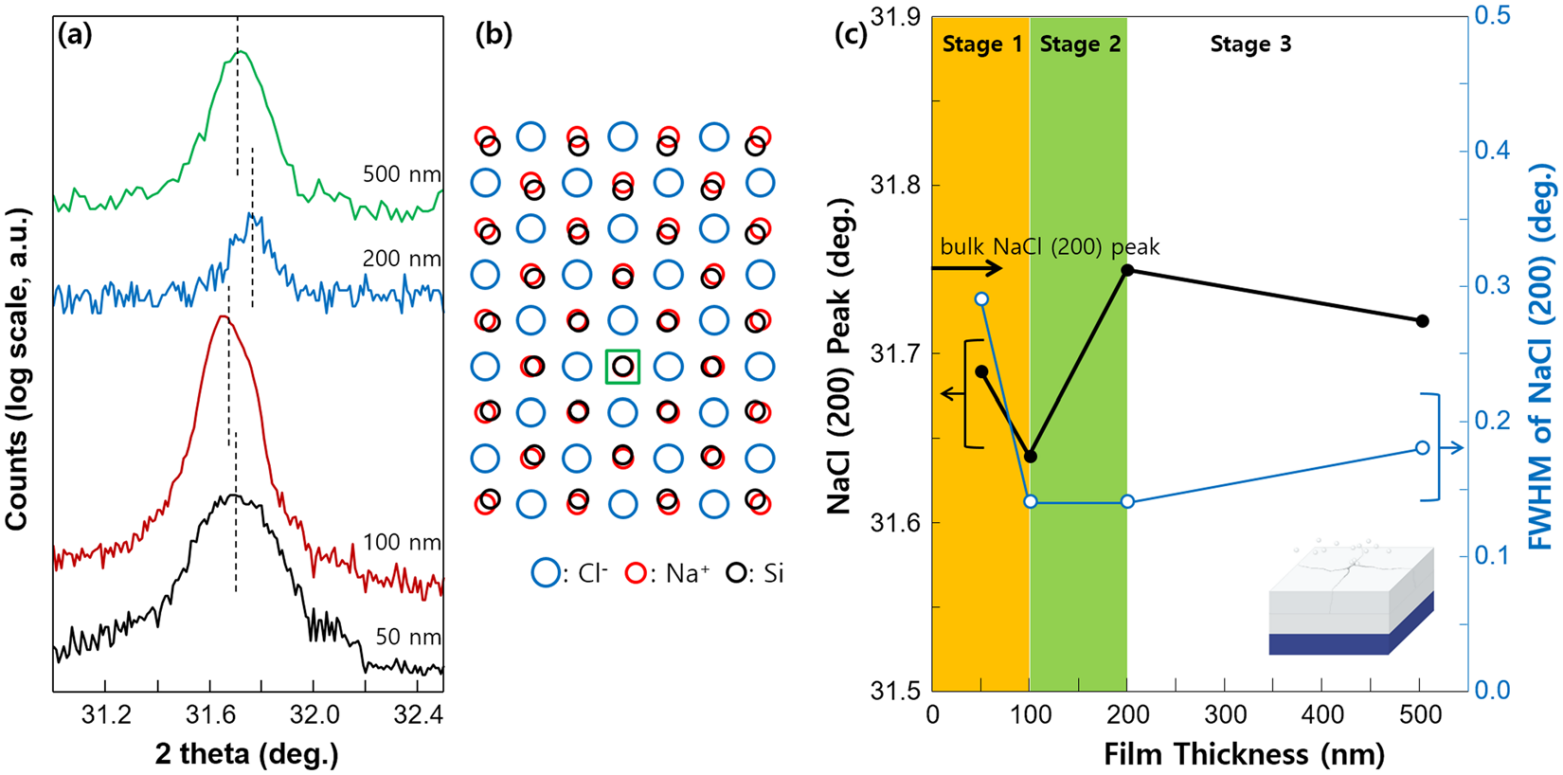
(**a**) Shift in the positions of NaCl(200) XRD peaks on Si(100) substrate. (**b**) Schematic representing the epitaxial growth of NaCl(100) on Si(100) substrate. (**c**) Peak positions and FWHM values of NaCl(200) peaks at different growth stages.

Epitaxial NaCl(200) films (thickness < 100 nm) on Si(100) substrate exhibited a shift in the 2θ position to smaller angles when compared to the bulk state (Fig. 3a and c). This is attributed to larger lattice size of NaCl (0.564 nm) than Si (0.543 nm) as shown in Fig. 3b, resulting in compressive strain in the in-plane direction and tensile strain in the out-of-plane direction. Accumulated strain in NaCl film was relaxed at film thicknesses > 200 nm, and position of the (200) peak approached that of the bulk state (Fig. 3a and c). The shift in the (200) peak to a smaller angle for thick NaCl film (thickness ≥500 nm) is due to compressive stress (in-plane direction) produced by NaCl atoms deposited between grain boundaries (schematic diagram in the inset of Fig. 3c). The evolution of strain between NaCl film and Si(100) substrate also affects the FWHM (full width at half maximum) of NaCl(200) peaks as shown in Fig. 3c. Diffraction angle and the FWHM of NaCl(200) peaks decreased with increase in film thickness in stage 1 (epitaxial growth stage). Epitaxial crystallinity of thin NaCl film on Si degrades (increase in the FWHM) from stage 2 (strain relaxation stage) by forming random orientations, and in stage 3, polycrystalline structure is formed^21^.

**Figure 4:**
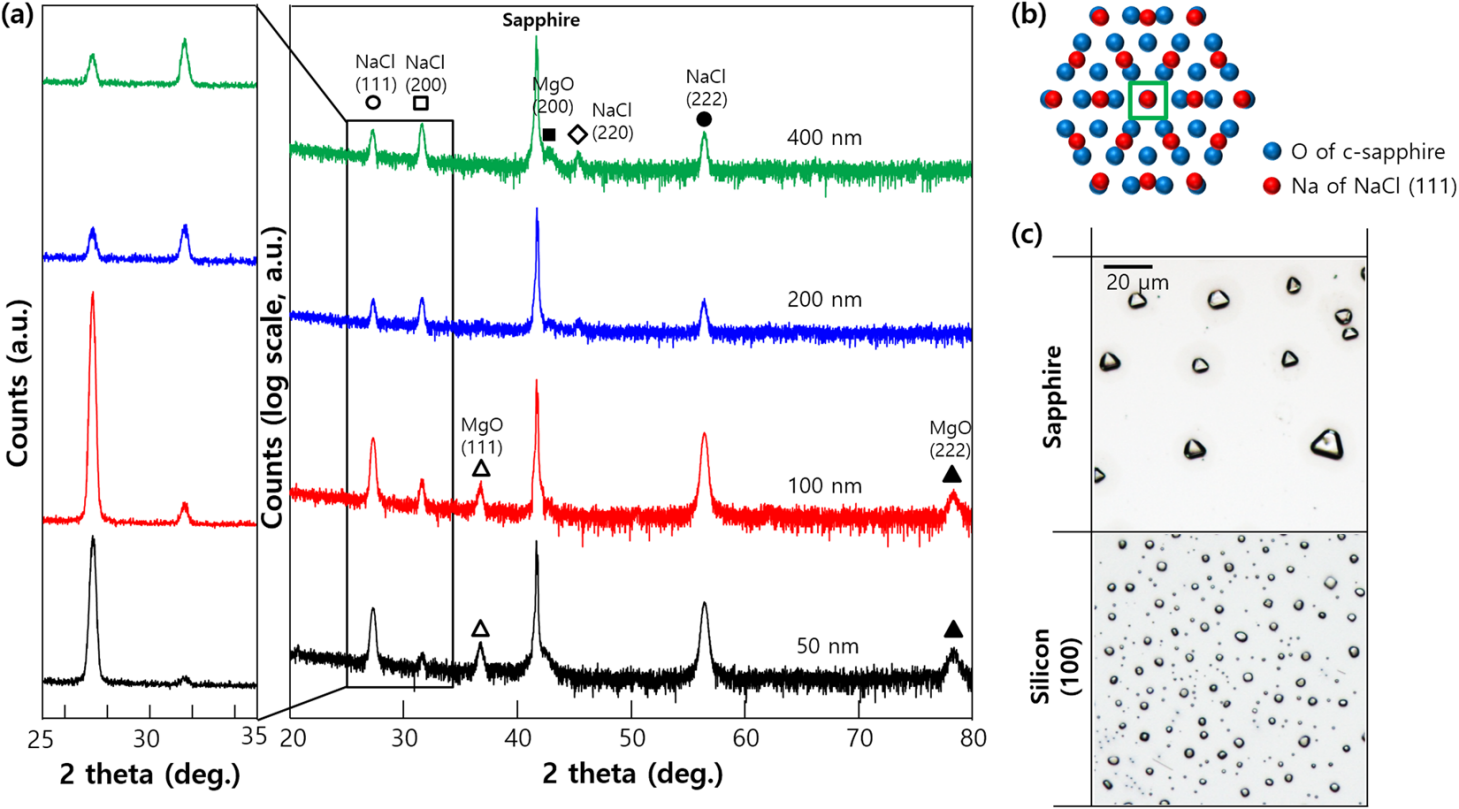
(**a**) XRD patterns of NaCl films grown on c-sapphire substrates at different film thicknesses. (**b**) Schematic of 2/3 domain matched epitaxial growth of NaCl(111) on c-sapphire substrate. (**c**) Optical microscopy images of recrystallized NaCl grains on c-sapphire (top) and Si(100) substrates (bottom).

To prepare epitaxial NaCl films with (111) orientation, we deposited NaCl on c-sapphire (Fig. 4a). NaCl films melt easily because of ambient water vapor, forming the low surface energy (200) plane of the rock-salt crystal system^22^. To protect epitaxial NaCl(111) orientation from water vapor, a 100-nm-thick magnesium oxide (MgO) film was deposited *in situ* on NaCl film^23, 24^. At thicknesses < 200 nm, intensity of the (111) peak was much higher than the (200) peak because the (111) crystal structure is preferred on the closed-packed c-sapphire substrate (considering the intensity ratio of (111) and (200) diffraction peaks per the JCPDS, i.e., 13:100). However, tendency for (111) preferred orientation of NaCl thin films on c-sapphire decreased significantly at thicknesses > 200 nm. NaCl(111) orientation may be grown on c-sapphire with 2/3 domain matching epitaxy, and domain mismatch is only −3.7% (Fig. 4b)^25, 26^. Epitaxial relations of NaCl(100)/Si(100) and NaCl(111)/c-sapphire were validated by analyzing shapes of reconstructed (dissolved by water vapor and recrystallized) NaCl grains (Fig. 4c). NaCl grains on c-sapphire substrate were triangular, whereas those on Si(100) substrate exhibited relatively rounded square shapes (from the smooth edge based on high surface energy at the NaCl crystal edge)^27^. From these results, it may be inferred that NaCl film has preferred orientation of (100) on Si(100) and (111) on hexagonal c-sapphire substrates.

**Figure 5:**
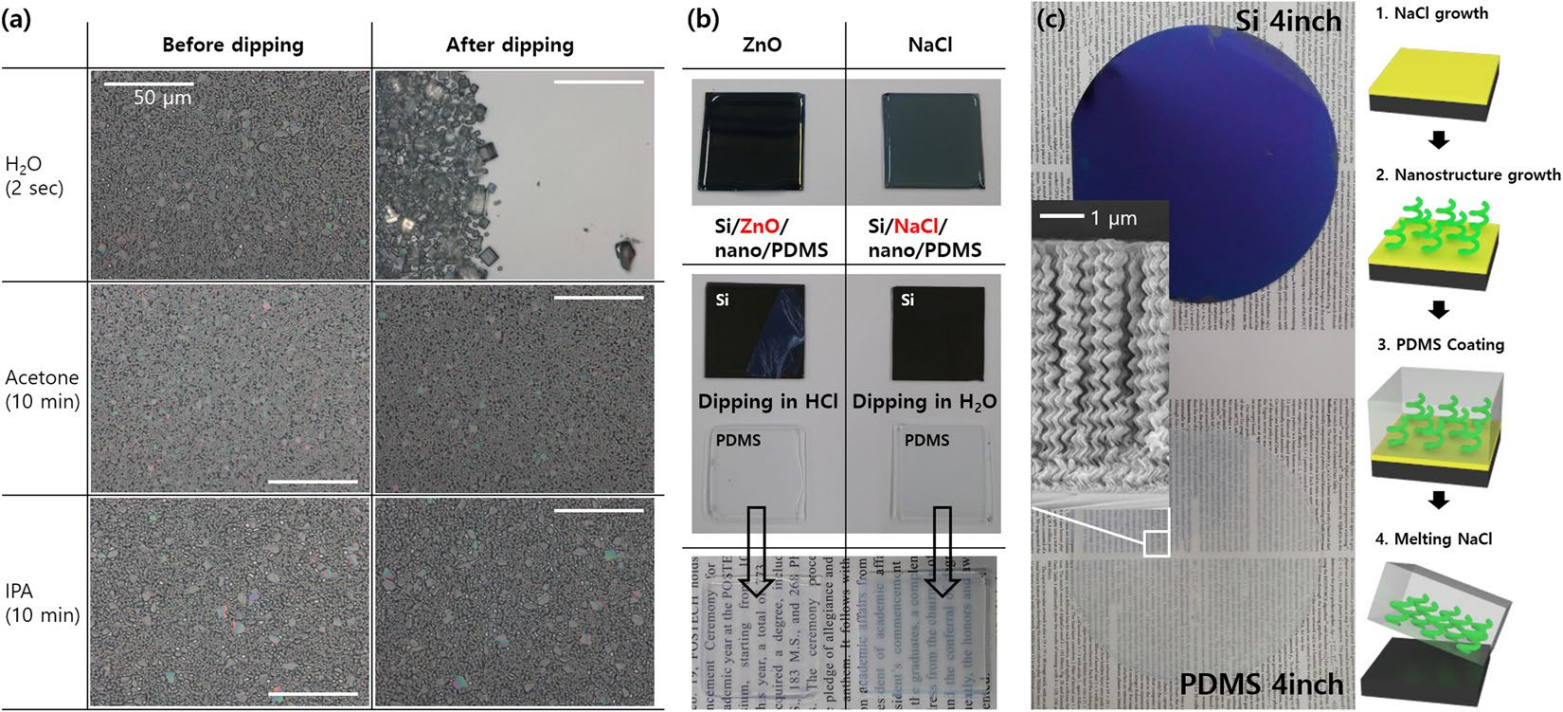
(**a**) Optical microscopy images of 500-nm-thick NaCl films before (left) and after (right) dipping in DI water, acetone, and IPA. (**b**) Photographs showing the transfer of WO_3_ on PDMS using ZnO (HCl solvent) and NaCl (water solvent) as the sacrificial layer. (**c**) Photographs and schematics showing the transfer of WO_3_ nanostructures from 4-inch Si wafer to PDMS using NaCl as the sacrificial layer. Inset is SEM images of WO_3_ nanostructures.

Figure 5a shows optical microscopy images of 500 nm-thick NaCl film on Si(100) substrate after dissolving in several solvents. NaCl film has higher solubility selectivity for water than for other solvents; NaCl films dissolved in water within two seconds resulting in recrystallized salt shapes, whereas the films were stable in acetone and isopropyl alcohol (IPA) even after 10 minutes^28^. NaCl thin film was applied as a water-soluble sacrificial layer for fabrication of flexible devices by transferring the WO_3_ nano-helix structures using PDMS supporting mold (Fig. 5b and c)^29^. Compared to conventionally used ZnO film, that may be dissolved only in acids, such as HCl, the NaCl sacrificial layer may be dissolved in water. Si substrate using NaCl as the sacrificial layer reveals a cleaner transfer yield compared to using ZnO. Water is safe and environment-friendly compared to HCl. As shown in Fig. 5c, this method may be successfully applied for large area processing of functional nanomaterials (4-inch wafer in this experiment).

**Figure 6:**
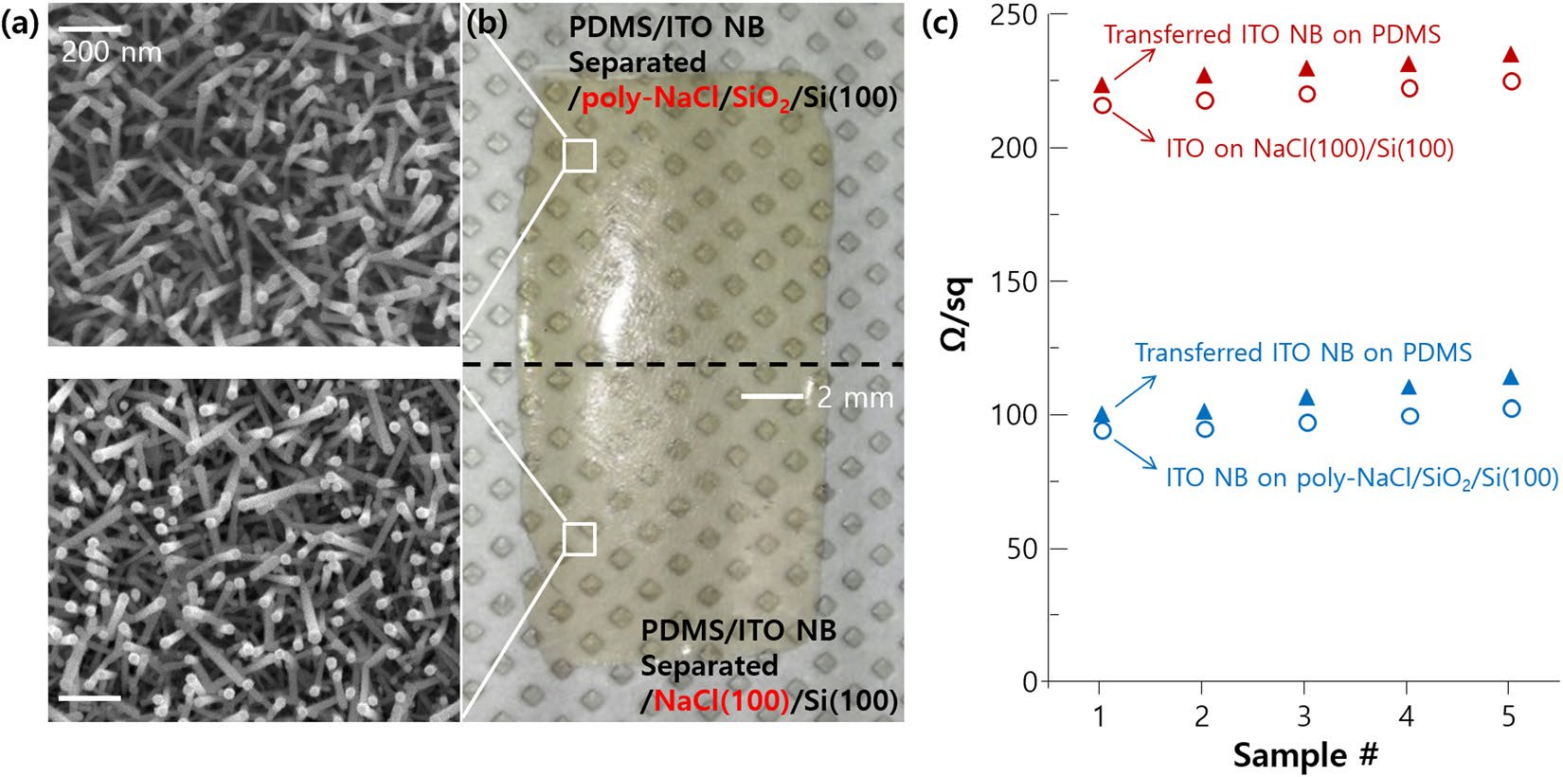
ITO NBs on poly-NaCl/SiO_2_/Si(100) and NaCl(100)/Si(100) (**a**) SEM images before PDMS transfer, (**b**) camera image after transfer. (**c**) Sheet resistivity of ITO NBs on poly-NaCl/SiO_2_/Si(100) and NaCl(100)/Si(100) before and after transfer. Each sample prepared 5 times for reproducibility.

We tested the effect of NaCl crystallinity on electrical properties of functional nano-materials (Sn doped In_2_O_3_ nano-branches: ITO NB) during the transfer process as shown in Fig. 6 ^30^. The ITO NBs grew vertically on NaCl(100)/Si(100) compare to that on poly-NaCl/SiO_2_/Si(100), resulting in relatively bright colors after transfer on PDMS by melting NaCl layers (Fig. 6a and b). Sheet resistivity of relative vertical ITO NBs reveals large values (220–230 Ω/sq) compare to randomly distributed ITO NBs (90–100 Ω/sq), and these values reveal similar tendency after transfer on PDMS. To reduce sheet resistivity of ITO NBs, we may 1) increase growth time to develop more branches for better connection between nanorods^31^, 2) anneal ITO NBs in a vacuum or inert atmosphere to increase additional oxygen vacancies for high electron concentration.

## Conclusion

In summary, we studied crystal structures of NaCl thin films prepared on Si(100) and c-sapphire substrates and applied film as a sacrificial layer in a new method developed for fabrication of flexible devices. Epitaxial preferred orientation of NaCl thin film may be controlled as (100) on Si(100) substrate and (111) on c-sapphire at thicknesses < 100 nm. As film thickness increases (thickness > 200 nm), accumulated stress between NaCl film and substrate is released by formation of random orientations. NaCl film has considerably high solubility selectivity for water, and may be applied as a water-soluble sacrificial layer for fabrication of flexible devices by transferring functional materials (not only the structural shape but also materials’ properties) using a PDMS supporting mold. Alternatively, polymer carrier film such as (poly(methyl methacrylate), polycarbonate, and polystyrene, etc) may be used as a transfer agent in this study. Polymer-coated functional materials may be separated from substrate by dissolving NaCl film in water and scooping to the target surface. After sufficient drying, polymer film may be removed with specific solvent. In this way, functional materials on NaCl may be transferred to the target surface freely. This procedure may be used in multiple research fields needing flexible functionality. Reconstructed NaCl nano or micro particles (cube, rods, branches, etc.) may be used as starting materials 1) for hollow ceramic shell structures and 2) three-dimensional graphene cage for application of biology (such as drug delivery) and energy related devices (such as cathode materials of Li-S and Na-S battery), resulting in interest from a range of scientific field readers from diverse communities.

## Methods

### Film growth and X-ray analysis

NaCl thin films were deposited by thermal evaporation. NaCl source material (99.5% purity; Sigma-Aldrich; product number S7653) was heated on a tungsten boat at 1.7 V and 45 A. N-type (100) silicon doped with arsenic and c-plane sapphire was used as starting substrate. Substrates were cleaned sequentially with acetone, ethyl alcohol, and DI (de-ionized) water. Native oxide on the Si wafer was removed by treating with hydrofluoric acid. Chamber pressure was −2 × 10^−5^ Torr during deposition, substrate was maintained at room temperature, deposition rate was 1.4 nm/s, and distance between the NaCl source and substrate was 32 cm.

To protect NaCl film from melting because of ambient water vapor, a MgO film was grown on NaCl film by electron-beam evaporation using high purity MgO pellets made by compressing and heating 99.995% purity MgO powder (Mitsubishi Materials Co.). Chamber pressure was −10^−6^ Torr during deposition, the substrate was maintained at room temperature, and deposition rate was 1 Å/s.

For comparative study of the NaCl sacrificial layer prepared in this study with conventionally used ZnO sacrificial layer, ZnO film was deposited using RF (radio frequency) magnetron sputtering. The ZnO target was prepared by sintering high-purity ZnO powder (99.9% purity; Aldrich Chemical Company). The sputtering system was pumped down to 8 × 10^−6^ Torr, and Ar gas was introduced to adjust working pressure to 2 mTorr. After pre-sputtering with Ar plasma for five minutes, 100-nm-thick ZnO films were deposited on Si substrates at RF power of 100 W. Substrate temperature was set to 400 °C, and distance between the target and substrate was 10 cm.

In-lab XRD studies were conducted using a powder X-ray diffractometer (18 kW; Mac Science; M18XHF22) equipped with a scintillation detector, and monochromatic Cu K*α* radiation was used for measurements. High-resolution XRD using synchrotron radiation was conducted at the 3C2 beamline at Pohang Accelerator Laboratory for azimuthal scan to measure epitaxial relationship.

### Growth of functional nanomaterials and transfer to PDMS

Tungsten oxide powder (WO_3_; Taewon Scientific Co.) was placed in a graphite crucible and subjected to electron beam evaporation. Prior to fabrication of WO_3_ nanohelix arrays, a thin WO_3_ film (thickness -50 nm) was deposited on the sacrificial layer (NaCl or ZnO) prepared on Si substrate by electron beam evaporation. Substrate was spun at 1 rpm for two seconds and stopped for 12 seconds with an oblique angle of 80°. A deposition rate of 3.5 Å/s was used to fabricate nano-helix arrays. Pressure in the chamber was maintained at 1.5 × 10^−6^ Torr during deposition of the WO_3_ film. As-deposited samples were annealed at 500 °C for one hour.

Self-assembled ITO nano-branches were fabricated by a conventional electron beam evaporation method^30^. ITO pellet sources were made by tin doped (10%) indium oxide powder (99.99%, from Mining & Chemical Product in UK) after sintering at 1,100°C during 24 hours in an atmospheric environment. ITO nano-branches were grown by evaporating pellets at a rate of 1 nm s^−1^. Chamber pressure was maintained at approximately 10^−5^ Torr during deposition and substrate temperature was held at 400°C. Thickness of ITO film with nano-branches was approximately 770 nm.

Mixture of PDMS polymer and curing agent in weight ratio of 10:1 was placed on the functional nanostructure grown on NaCl or ZnO thin film and heated to 90 °C for three minutes. After curing PDMS, samples were dipped in DI water (for NaCl film) or HCl (for ZnO film) to dissolve the sacrificial layer and separate it from Si substrate. Since NaCl is considerably vulnerable to water, the process should proceed as soon as possible after deposition. Also, the device should be a material that is insoluble in water because water may damage the device during transferring. DI water cleaning process is needed after dissolving NaCl thin film because NaCl particle may be recrystallized on the functional nanostructure. It could decrease electrical conductivity and transparency of transferred materials.

### Data availability

The data that support the findings of this study is available from the corresponding author upon request.
